# Application of polymer-coated *Macadamiaintegrifolia* nutshell biomass impregnated with palladium for chromium(VI) remediation

**DOI:** 10.1038/s41598-021-03473-8

**Published:** 2021-12-17

**Authors:** Malvin Moyo, Sekomeng Johannes Modise, Vusumzi Emmanuel Pakade

**Affiliations:** 1 Department of Chemistry, Vaal University of Technology, Vanderbijlpark, 1911 South Africa; 2 Department of Applied Chemistry, National University of Science and Technology, Bulawayo, Zimbabwe

**Keywords:** Environmental sciences, Chemistry

## Abstract

Freely suspended and porous basket restrained granules of palladium nanoparticles supported on polymer-grafted *Macadamia* nutshell biomass (Pd@Polym-MNS) composite were used for the treatment chromium(VI)-containing water. In the presence of formic acid, the Pd@Polym-MNS demonstrated its activity in the adsorption-reduction-based conversion of noxious chromium(VI) to less toxic chromium(III) with a low activation energy of 13.4 kJ mol^–1^, ΔH^0^ (+ 10.8 kJ mol^–1^), ΔS^0^ (−270.0 J mol^–1^ K^–1^), and ΔG^0^ (+ 91.3 to + 98.0 kJ mol^–1^) indicated the exothermic, endergonic and non-spontaneous nature of the catalytic redox reaction. In addition to facilitating easy recovery, rinsing, and reuse, restraining the Pd@Polym-MNS in the basket reactor helped maintain the integrity of the catalysts by preventing violent collisions of suspended granules with the mixing apparatus and the walls of the reaction vessel. Whereas the pseudo-first-order rate constant was recorded as 0.157 min^–1^ upon initial use, values of the mean and relative standard deviation for the second, third and fourth consecutive uses were found to be 0.219 min^–1^ and 1.3%, respectively. According to a response surface methodological approach to batch experimentation, the initial concentration of chromium(VI) and catalyst dosage had the greatest impact on the redox reaction rate, accounting for 85.7% and 11.6% of the variability in the value of the pseudo-first-order rate constant, respectively. Mutually beneficial effects of the combinations of high formic acid and low chromium(VI) concentration, high temperature and catalyst dosage as well as high formic acid and catalyst dosage were recorded.

## Introduction

From a health perspective, the presence of hexavalent chromium [Cr(VI)] in water is of primary concern owing to its cancer-causing effects on animal cells^1^. The toxicity of Cr(VI) is exacerbated by the ability of chromate ions, which structurally resemble sulfate ions, to be easily transported into cells through sulfate transporters^2^. Moreover, cell membranes are less permeable to trivalent chromium [Cr(III)] species^3^ rendering Cr(III) much less toxic than Cr(VI). Techniques based on adsorption^4–6^, solvent extraction^7,8^, and membrane filtration^9,10^ have proved to potentially provide feasible options for the removal of Cr(VI) from water. However, the main drawback associated with the removal techniques is that the pollutant often remains in its toxic hexavalent state, thereby retaining some degree of threat to the environment upon disposal of the used adsorbent, the retentate from membrane filtration, and the concentrate obtained after solvent recovery following solvent extraction. Several emerging strategies for the remediation of Cr(VI)-contaminated water, therefore, involve the transformation of Cr(VI) to Cr(III), which, for easier containment, can be precipitated as hydroxides^11^. Photocatalyzed Cr(VI) reduction, which allows the use of water as the reducing agent thereby eliminating the need for chemical reductants, has sparked great interest and research effort^12–15^. However, the costs linked to either the generation of ultraviolet light or the development of photocatalysts with high activity in the visible light (solar) region, continue to plague the use of photocatalysis in Cr(VI) remediation.

The reduction of the Cr(VI) in, for example, aqueous hydrogen chromate (HCrO_4_^–^) ions by formic acid (HCOOH), which proceeds as per Eq. (1)^16,17^, is associated with positive electrode potentials indicating thermodynamic feasibility. However, the Cr(VI)-HCOOH redox reactions are still hampered by high activation barriers, which necessitate the use of a catalyst^18^. Heterogeneous catalysis in the Cr(VI)-HCOOH redox system often exploits the decarboxylation of HCOOH, which provides adsorbed hydrogen atoms (H_ads_) in accordance with Eq. (2) ^19^. The adsorbed hydrogen atoms subsequently donate their electrons to adsorbed Cr(VI) ions as exemplified by Eq. (3) ^20,21^. Recent reports detail the catalytic activity of materials based on different precious metals in the dehydrogenation, including gold, silver, rhodium, platinum, and palladium^22–26^. Although the activity of less costly alternatives such as nickel^16,20^, cobalt^27^, and sulfur^28^ has previously been reported, their use is impeded by easy leaching in the acidic HCOOH solution, resulting in poor reusability.

1 $${{\text{2HCrO}}_{4}}^{-} + \text{3HCOOH+} \,{\text{8H}}^{+} \, \rightarrow\, \text{2}{\text{Cr}}^{3+} \, + {\text{3CO}}_{2}\, +\,{\text{8H}}_{2}{\text{O}} \quad \,\,\, {\text{E}}^{\theta}= 1.48\text{ V} \,$$

2 $$\text{HCOOH } \, \rightarrow\, {\text{CO}}_{2}\, +\,{\text{2H}}_{\text{ads}}$$

3 $${{{\text{HCrO}}_{4}}^{-}}_{\text{ads}}\, +\,{\text{3H}}_{\text{ads}}\, +\,{\text{4H}}^{+} \, \rightarrow\, {{\text{Cr}}^{3+}}_{\text{ads}} \, + {\text{4H}}_{2}{\text{O}}$$

4 $${{\text{HCrO}}_{4}}^{-}\, +\,{\text{Fe}}^{0}\, +\,{\text{7H}}^{+} \, \rightarrow\, {\text{Cr}}^{3+} \, + \text{ } {\text{Fe}}^{3+}\, +\,{\text{4H}}_{2}{\text{O}}$$

5 $${{\text{HCrO}}_{4}}^{-}\text{ + 3}{\text{Fe}}^{2+}\, +\,{\text{7H}}^{+} \, \rightarrow\, {\text{Cr}}^{3+} \, + \text{ 3} {\text{Fe}}^{3+}\, +\,{\text{4H}}_{2}{\text{O}}$$

6 $$\text{HCOOH }\,\rightarrow\, {\text{CO}}_{2}\, +\,{\text{H}}_{2}$$

Based on the relatively low cost of iron-based materials, several studies suggest that the use of zerovalent or divalent iron as reducing agents for the transformation of Cr(VI) to Cr(III) provides viable options for Cr(VI) remediation^29–36^. However, as illustrated by Eqs. (4) and (5), the resulting waste contains aqueous iron(III) species, which also precipitate alongside the Cr(III) thereby yielding large volume of sludge thus limiting the efficiency and applicability of iron-based chemical reduction techniques. Moreover, due to contamination by pure and mixed hydroxides, Fe(OH)_3_ and Cr_x_Fe_1−x_(OH)_3_, the precipitated chromium(III) hydroxide has lower value than purer forms formed by precipitation from wastes devoid of iron(III) species. Since the use of HCOOH as the reducing only produces carbon dioxide and water, waste solutions devoid of iron(III) species can be produced. Furthermore, excess HCOOH in the reaction mixture can be decomposed on the surface of the catalyst to liberate hydrogen gas (H_2_), an environmentally friendly fuel, in accordance with Eq. (6)^37,38^, and the decomposition of excess HCOOH lowers the acidity of the solution prior to the precipitation of chromium(III) hydroxide. Therefore, the elevated costs associated with the precious metal-based catalysts used in the Cr(VI)-HCOOH redox system can be offset by the value of the hydrogen gas and the pure chromium(III) hydroxide that can be produced via subsequent treatment with sodium hydroxide, calcium hydroxide or magnesium oxide^39,40^.

Even though the use of nanoparticles results in enhanced catalysis, the recovery of the nanoparticles for reuse is often difficult and poses the risk of release into the environment together with the treated water. Therefore, the catalytic nanoparticles used in the HCOOH-mediated reduction of Cr(VI) have been immobilized on powders, granules, and films^16,17,41–47^. Despite several materials being used as supports for catalytic nanoparticles used in the HCOOH-mediated reduction of Cr(VI), the use of lignocellulosic material supports is scarcely reported in the literature. Following the synthesis and characterization of palladium nanoparticles dispersed on polymer-grafted *Macadamia* nutshell (Pd@Polym-MNS) granules in our previous work^48^, we have restrained the Pd@Polym-MNS granules in a porous stainless steel basket. The practical implication of restraining the catalytic Pd@Polym-MNS granules in a porous basket is that it enables easy application and recovery by immersion and retrieval, respectively, without the need for elaborate, time consuming steps in industrial wastewater treatment processes.

## Materials and methods

### Materials

The preparation and characterization of the composite comprising polymer-coated *Macadamia integrifolia* nutshell biomass with embedded Pd nanoparticles (Pd@Polym-MNS) were as described in our recent report ^48^. Waste *Macadamia* nutshells were kindly donated by the Eastern Produce Estates SA (Pty) Ltd, Tzaneen, South Africa and all experiments were performed in accordance with relevant guidelines and regulations. All reagents were sourced from Johannesburg, South Africa. Potassium dichromate (K_2_Cr_2_O_7_) and formic acid (HCOOH, 85 wt%) were purchased from Merck Chemical Company, and Rochelle Chemicals, respectively. Sodium sulfate (Na_2_SO_4_), sodium chloride (NaCl), and sodium nitrate (NaNO_3_) were obtained from Sigma-Aldrich. Sulfuric acid (H_2_SO_4_, 98% wt%) and hydrochloric acid (HCl), 32% (w/w) were purchased from Associated Chemical Enterprises. A stock solution of Cr(VI) was prepared by dissolving the K_2_Cr_2_O_7_ in deionized water obtained from a Milli-Q water system (Milli-Q Direct, with 18.2 MΩ cm resistivity, Merck Millipore Corporation, USA).

### Cr(VI) conversion experiments

The experiments to assess the conversion of Cr(VI) by reaction with HCOOH were carried out in three batches. In the first batch of experiments, mixed Cr(VI)-HCOOH solutions were prepared by diluting mixtures of the Cr(VI) stock solution, H_2_SO_4_ and HCOOH with deionized water. Thereafter, the solution was incubated at a specific temperature, the Pd@Polym-MNS was added, and the suspension was magnetically stirred throughout the experiment. A porous basket reactor comprising a 40 mesh stainless steel cylindrical basket into which Pd@Polym-MNS was weighed before covering with a shafted lid was used in the second and third experimental batches. In the second batch of Cr(VI) conversion experiments, the Pd@Polym-MNS-loaded basket was immersed in an incubated mixed Cr(VI)-HCOOH solution prepared by the dilution of mixtures of the Cr(VI) stock solution, HCOOH, H_2_SO_4_, NaCl, and NaNO_3_ with tap water. The basket was either kept stationary as the solution was stirred using an overhead paddle or the solution was stirred by rotation of the basket reactor on a dissolution testing apparatus (Vision G2 Classic 6, Teledyne Hanson Research, Chatsworth, CA, USA). In order to facilitate the analysis of the combined influence of initial Cr(VI) concentration (*X*_*1*_), initial HCOOH concentration (*X*_*2*_), Pd@Polym-MNS dose (*X*_*3*_), and temperature (*X*_*4*_) on the rate of Cr(VI) conversion, the third batch of Cr(VI) conversion experiments was conducted as per a 2^4^ factorial central composite experimental design with the coded factor levels and values of the input or independent variables presented in Table 1.

**Table 1:** Input variable codes, levels, and values of the central composite design-based experiment.

Input variables	Code	Variable levels and values				
		–2	–1	0	+ 1	+ 2
Initial Cr(VI) concentration, *[Cr(VI)]*_*0*_, (mM)	*X*_*1*_	0.5	1.5	2.0	2.5	3.0
Initial HCOOH concentration, *[HCOOH]*_*0*_, (mM)	*X*_*2*_	50	100	200	300	500
Pd@Polym-MNS dose, *Dose*, (g L^–1^)	*X*_*3*_	0.5	1.0	2.0	3.0	5.0
Temperature, *T*, (°C)	*X*_*4*_	15	25	35	45	55

Using the rate constant of the model providing the best fit to the Cr(VI) conversion kinetics as the response or dependent variable (*Y*), the relationship between the response and input variables was modeled using a quadratic equation expressed as:

7 $${\text{Y}}=\beta_{0}\, +\,\beta_{1}{{\text{X}}}_{1}\, +\,\beta_{2}{{\text{X}}}_{2}\, +\,\beta_{3}{{\text{X}}}_{3}\, +\,\beta_{4}{{\text{X}}}_{4}\, +\,\beta_{12}{{\text{X}}}_{1}{{\text{X}}}_{2}\, +\,\beta_{13}{{\text{X}}}_{1}{{\text{X}}}_{3}\, +\,\beta_{14}{{\text{X}}}_{1}{{\text{X}}}_{4}\, +\,\beta_{23}{{\text{X}}}_{2}{{\text{X}}}_{3}\, +\,\beta_{24}{{\text{X}}}_{2}{{\text{X}}}_{4}\, +\,\beta_{34}{{\text{X}}}_{3}{{\text{X}}}_{4}\, +\,\beta_{11}{{\text{X}}_{1}}^{2}\, +\,\beta_{22}{{\text{X}}_{2}}^{2}\, +\,\beta_{33}{{\text{X}}_{3}}^{2}\, +\,\beta_{44}{{\text{X}}_{4}}^{2} \,$$

where *Y* represents the predicted response; *β*_*0*_ the offset term; *β*_*1*_, *β*_*2*_, *β*_*3,*_ and *β*_*4*_ the linear coefficients; *β*_*12*_, *β*_*13*_, *β*_*14*_, *β*_*23*_, *β*_*24*_ and *β*_*34*_ the interaction coefficients; and *β*_*11*_, *β*_*22*_, *β*_*33,*_ and *β*_*44*_ the quadratic coefficients. *X*_*1*_, *X*_*2*_, *X*_*3,*_ and *X*_*4*_ represent the input variables as defined in Table 1^49^. Using Statistica software (Version 10.0, StatSoft, Inc., Tulusa, OK, USA), Eq. (7) was fitted to the experimental data, and surface plots were created to evaluate the combined effects of the input variables.

At specific times during the course of each Cr(VI) conversion experiment, a 3.5 mL sample of the working solution was transferred into an optical quartz cell with a 1 cm path length and its ultraviolet–visible (UV–Vis) spectral absorption was recorded on a UV–Vis spectrophotometer (Cary 60, Agilent Technologies, Santa Clara, CA, USA) over a wavelength range of 300 nm to 500 nm at a scanning rate of 1200 nm min^–1^ with increments of 2 nm and a step time of 0.10 s. Immediately afterward (approximately 2 min after sampling), the sample was returned to the reaction mixture. The evolution of Cr(VI) concentration was monitored by measurement of the absorbance of the peaks at the characteristic wavelengths between 350 and 375 nm^50^. In accordance with the Beer-Lambert Law, which states that spectral absorbance is directly proportional to concentration, the absorbance of the Cr(VI) solutions at the beginning of each Cr(VI) conversion experiment and at any time *t* thereafter, denoted *A*_*0*_ and *A*_*t*_ (arbitrary units), respectively, was related to the Cr(VI) concentration at the beginning of each experiment and at any time *t* during the experiment, denoted where *[Cr(VI)]*_*0*_ and *[Cr(VI)]*_*t*_ (mmol L^–1^), respectively, in accordance with Eq. (8). The fraction of Cr(VI) that remained in the solution any time during the experiment, designated *Residual Cr(VI)* (%), was calculated using Eq. (9).

8 $$\frac{{\text{A}}_{\text{t}} \, }{{\text{A}}_{0}} = \frac{{\text{[Cr(VI)]}}_{\text{t}} \, }{{\text{[Cr(VI)]}}_{0}}$$

9 $$\text{Residual Cr(VI)} = \frac{{\text{A}}_{\text{t}} \, }{{\text{A}}_{0}} \times {100\%}$$

The possibility of palladium leaching during the treatment of the acidic mixed Cr(VI)-HCOOH solutions was evaluated by analysis of final solutions using an inductively coupled plasma optical emission spectrophotometer (iCAP 7000, Thermo Fisher Scientific, Waltham, MA, USA).

### Kinetic and thermodynamic data analysis

The rate law for Cr(VI) conversion by reaction with HCOOH is represented by Eq. (10) where *v* (mmol L^–1^ min^–1^) is the rate to Cr(VI) conversion, *[Cr(VI)]* (mmol L^–1^) and *[HCOOH]* (mmol L^–1^) are the concentrations of Cr(VI) and HCOOH, respectively, *k* (mmol^(1 – *a* – *b*)^ L^(*a* + *b* – 1)^ min^–1^) is the rate constant, and *a* (dimensionless) and *b* (dimensionless) represent the order of reaction with respect to Cr(VI) and HCOOH, respectively. Since an excess amount of HCOOH was used throughout the Cr(VI) conversion experiments, changes in *[HCOOH]* can be considered insignificant so that *k[HCOOH]*^*a*^ constitutes a new rate constant referred to as the apparent rate constant, *k*_*app*_ (mmol^(1 – *b*)^ L^(*b *– 1)^ min^–1^), and the rate law for Cr(VI) conversion by reaction with HCOOH can be expressed as Eq. (11).

10 $${\text{v}} = \frac{{\text{d[Cr(VI)]}}}{{\text{dt}}} = - {\text{k}}[{\text{HCOOH}}]^{\text{a}}[{\text{Cr(VI)}}]^{\text{ b}}$$

11 $${\text{v}} = \frac{{\text{d[Cr(VI)]}}}{{\text{dt}}} = - {\text{k}}_{\text{app}}[{\text{Cr(VI)}}]^{\text{b}}$$

The kinetics of Cr(VI) conversion were profiled using the pseudo-zeroth-order, pseudo-first-order and pseudo-second-order models associated with the integrated rate laws expressed as Eqs. (12), (13) and (14) where the orders of reaction with respect to Cr(VI) are *b* = 0, *b* = 1 and *b* = 2 in addition to *k*_*0,app*_ (mmol L^–1^ min^–1^), *k*_*1,app*_ (min^–1^) and *k*_*2,app*_ (mmol^–1^ L min^–1^) representing the apparent pseudo-first-order rate constant, respectively.

12 $$\mathrm{Pseudo-zeroth-order}:\quad\,\,\frac{{\text{[Cr(VI)]}}_{\text{t}} \, }{{\text{[Cr(VI)]}}_{0}} = {1 - }\frac{{\text{k}}_{\text{0,app}}\text{t }}{{\text{[Cr(VI)]}}_{0}}$$

13 $$\mathrm{Pseudo-first-order}: \quad\,\,\frac{{\text{[Cr(VI)]}}_{\text{t}} \, }{{\text{[Cr(VI)]}}_{0}} = {\text{exp}}\left(-{\text{k}}_{\text{1,app}}{\text{t}}\right)$$

14 $$\mathrm{Pseudo-second-order}: \quad\,\,\frac{{\text{[Cr(VI)]}}_{\text{t}} \, }{{\text{[Cr(VI)]}}_{0}} \, = \, \frac{{1} \, }{{\text{[Cr(VI)]}}_{0}{{\text{k}}}_{\text{2,app}}{\text{t}}{ + 1}}$$

The thermodynamic data were analyzed by plotting the first-order rate constant versus temperature and carrying out nonlinear regression fitting of the Arrhenius and Eyring equations expressed as Eq. (15) and Eq. (16), respectively, where *k*_*app*_ (mmol^(1 – *b*)^ L^(*b* –1)^ min^–1^) is the rate constant, *A* (mmol^(1 – *b*)^ L^(*b* –1)^ min^–1^) is the pre-exponential factor, *E*_*a*_ (J mol^−1^) is the apparent activation energy of the reaction, *R* is the universal gas constant (8.3145 J K^−1^ mol^−1^), *T* (K) is the temperature, *E*_*y*_ is an Eyring equation constant (1 mmol^(1 – *b*)^ L^(*b* –1)^ min^–1^ where *b,* the order of reaction with respect to Cr(VI) is 0, 1 or 2), *k*_*B*_ is Boltzmann’s constant (1.3807 × 10^–23^ J K^–1^), *h* is Planck’s constant (6.6262 × 10^–34^ J s), *ΔS*^*‡*^ (J mol^–1^ K^−1^) is the change in entropy of activation and *ΔH*^*‡*^ (J mol^–1^) is the change in enthalpy of activation^51,52^. Values of the change in Gibbs free energy of activation, *ΔG*^*‡*^ (J mol^–1^), at each temperature were further calculated as expressed in Eq. (17).

15 $$\mathrm{Arrhenius}: \quad {\text{k}}_{\text{app}}\, = \, \, {\text{A}}\text{ exp}\left({-}\frac{{\text{E}}_{\text{a}}}{\text{RT}}\right)$$

16 $$\mathrm{Eyring}:\quad {\text{k}}_{\text{app}}\, = \,{\text{E}}_{\text{y}} \, \frac{{\text{k}}_{\text{B}}{\text{T}}}{\text{h}}\text{ exp}\left(\frac{{\Delta\text{S}}^{\ddagger}}{\text{R}}{ - }\frac{{\Delta\text{S}}^{\ddagger}}{\text{RT}} \right)$$

17 $${\Delta\text{G}}^{\ddagger}\, = \, \, {\Delta\text{H}}^{\ddagger}\text{ - }{{\text T} \Delta {\rm S}}^{\ddagger}$$

Using nonlinear regression based on minimization of the Chi-square statistic, denoted *χ*^*2*^ (dimensionless), curves relating *[Cr(VI)]*_*t*_/*[Cr(VI)]*_*0*_ to *t*, and *k*_*app*_ to *T*, were fitted to the kinetic and thermodynamic experimental data, respectively. The corresponding values of the coefficient of determination, *R*^*2*^ (dimensionless), were also calculated. The values of *χ*^*2*^ and *R*^*2*^ were determined using Eqs. (18) and (19), respectively, expressed as:

18 $${\chi }^{2}\, = \,\sum_{\text{i = }{1}}^{\text{n}}\frac{{\left({\text{x}}_{\text{i,expt}} \, {-} \, {\text{x}}_{\text{i,mod}}\right)}^{2}}{{\text{x}}_{\text{i,mod}}}$$

19 $${\text{R}}^{2}\text{ = 1 } {-} \, \frac{\sum_{\text{i } = \text{ 1}}^{\text{n}}{\left({\text{x}}_{\text{i,expt}} \, {-} \, {\text{x}}_{\text{i,mod}}\right)}^{2}}{\sum_{\text{i } = \text{ 1}}^{\text{n}}{\left({\text{x}}_{\text{i,expt}} \, {-} \, {\stackrel{\mathrm{-}}{\text{x}}}_{\text{i,expt}}\right)}^{2}}$$

where *x*_*i,expt*_ represents the measured or experimentally determined values of *[Cr(VI)]*_*t*_/*[Cr(VI)]*_*0*_ or *k*_*app*_, *x*_*i,mod*_ the model-predicted values of *[Cr(VI)]*_*t*_/*[Cr(VI)]*_*0*_ or *k*_*app*_, ${\stackrel{\mathrm{-}}{\text{x}}}_{\text{i,expt}}$ the average of the experimentally determined *[Cr(VI)]*_*t*_/*[Cr(VI)]*_*0*_ or *k*_*app*_ measurements, and *n* is the number of measurements^53^. Using the Solver Add-in of Microsoft Excel software, the curve fitting process involved a trial-and-error-based iterative method wherein the parameters of each model equation were adjusted until the lowest value of *χ*^*2*^ was attained.

## Results and discussion

### Efficacy of the HCOOH-Cr(VI) redox system in Cr(VI) conversion

**Figure 1:**
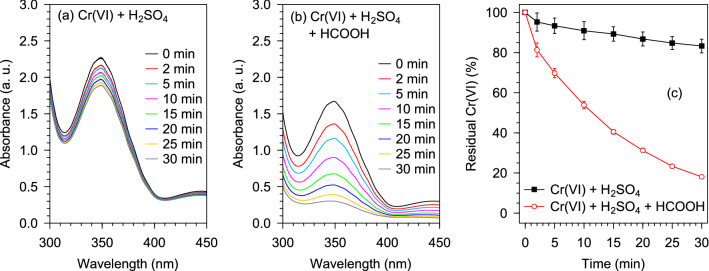
Evolution of the UV–Vis spectra of Pd@Polym-MNS-treated Cr(VI) aqueous solution with added **(a)** H_2_SO_4_ only (control), and **(b)** H_2_SO_4_ and HCOOH. **(c)** Corresponding evolution of residual Cr(VI) in solution. Fixed experimental conditions: V = 250 mL; Pd@Polym-MNS dose = 2.0 g L^–1^; [Cr(VI)]_0_ = 2.0 mM; [H_2_SO_4_]_0_ = 1875 mM; [HCOOH]_0_ = 1000 mM; T = 25 °C.

In order to emphasize the efficacy of the reducing agency of HCOOH in the Pd@Polym-MNS facilitated conversion of Cr(VI) to Cr(III) ions, two varieties of Cr(VI) solution were treated with the Pd@Polym-MNS. The first variety of solution contained 0.6 mM Cr(VI),1875 mM H_2_SO_4_ and 1000 mM HCOOH whereas the second contained only Cr(VI) (0.6 mM) and H_2_SO_4_ (1875 mM). On both occasions, function of the sulfate ions supplied by the H_2_SO_4_ was to mask the protonated and polar functional groups on the Pd@Polym-MNS surfaces thereby hindering the adsorption of Cr(VI) ions. Having masked the protonated and polar functional groups, the principal mechanisms responsible observed decreases in Cr(VI) concentration were redox reaction involving Cr(VI) ions adsorbed on the exposed surfaces of the Pd particles dispersed on the Pd@Polym-MNS. As shown in Fig. 1, the spectra of the two solutions obtained at different times during the respective experiments exhibited intense peaks near 350 nm assigned to oxygen-to-Cr(VI) charge transfer transition in Cr(VI) ions^54^.

**Figure 2:**
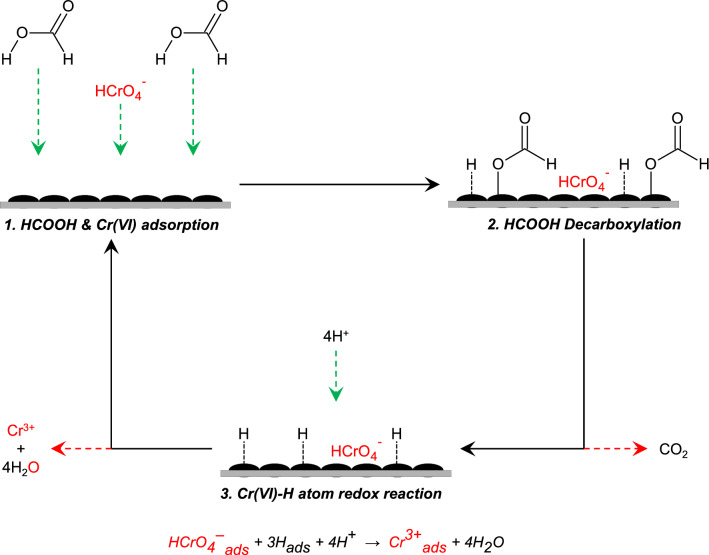
Tentative mechanism for Cr(VI) reduction by HCOOH over Pd@Polym-MNS.

Whether or not HCOOH was included in the Pd@Polym-MNS-treated solution, there was a gradual decrease in the characteristic peak attesting to the occurrence of Cr(VI) reduction on the Pd@Polym-MNS surfaces. However, the extent of Cr(VI) reduction appeared to be much greater in the solution that contained HCOOH, which, after 30 min of contact with the Pd@Polym-MNS, had a residual Cr(VI) proportion of 18.1% in comparison to 83.3% noted for the solution that did not contain HCOOH (Fig. 1c). The less effective reduction of Cr(VI) in the absence of HCOOH was ascribed to less potent reducing agency of oxidizable organic functional groups of the Pd@Polym-MNS, chiefly primary amines (–NH_2_) and hydroxyls (–CH_2_OH), which were oxidized upon redox reaction with the Cr(VI) ions^48^. In addition to having lower potency in comparison to that of HCOOH, the oxidizable organic functional groups were present in lower concentrations than the HCOOH. On account of the Cr(VI) ions undergoing redox reaction with adsorbed hydrogen atoms from the degradation of HCOOH on the Pd@Polym-MNS surface as illustrated in Fig. 2 followed by the desorption of the nascent Cr(III) ions, which regenerated the Cr(VI) adsorption sites for further uptake of Cr(VI) ions^17^, the extent Cr(VI) adsorption from the solution containing HCOOH was higher. Consequently, the rate at which the intensity of the characteristic peak decreased was higher when the HCOOH-containing solution was treated with the Pd@Polym-MNS.

### Thermodynamics of Pd@Polym-MNS-facilitated Cr(VI) conversion

**Figure 3:**
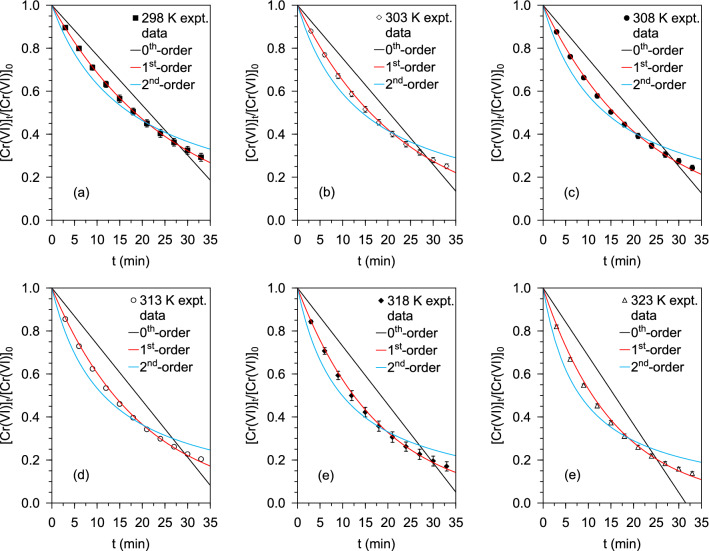
Kinetic plots of remaining fraction of Cr(VI) versus reaction time for Cr(VI) reduction by HCOOH over Pd@Polym-MNS at different temperatures: **(a)** 298 K, **(b)** 303 K, **(c)** 308 K, **(d)** 313 K, 318 K and 323 K. Fixed experimental conditions: V = 100 mL; Pd@Polym-MNS dose = 2.0 g L^–1^; [Cr(VI)]_0_ = 0.6 mM; [HCOOH]_0_ = 75 mM.

The thermodynamic aspects of the catalytic Cr(VI) conversion reaction were assessed by treatment of aliquots of a mixed Cr(VI)-HCOOH solution containing 0.6 mM Cr(VI) and 75 mM HCOOH with Pd@Polym-MNS incubated at different temperatures. The corresponding kinetic plots of *[Cr(VI)]*_*t*_/*[Cr(VI)]*_*0*_ versus *t* with fitted equations of the pseudo-zeroth-order, pseudo-first-order, and pseudo-second-order kinetic models are displayed in Fig. 3. As shown by the *Cr(VI)]*_*t*_/*[Cr(VI)]*_*0*_ versus *t* plots, and in accordance with production of the lowest values of *χ*^*2*^ plus the highest *R*^*2*^ values in the 0.9986 to 0.9999 range (Table 2), the kinetic data were well simulated by the pseudo-first-order kinetic model, which matched previous reports^17,43,48,55,56^. Therefore, the rate constant of the pseudo-first-order model equation was used as the apparent rate constant (*k*_*app*_) for evaluation of the thermodynamics of the Cr(VI) conversion process.

**Table 2:** Fitted pseudo-first-order, pseudo-first-order, pseudo-second-order rate constant and error statistic values for the HCOOH-mediated Cr(VI) conversion on Pd@Polym-MNS at different temperatures.

T (K)	Pseudo-zeroth-order			Pseudo-first-order			Pseudo-second-order		
	*k*_*0*_	*χ*^*2*^	*R*^*2*^	*k*_*1*_	*χ*^*2*^	*R*^*2*^	*k*_*2*_	*χ*^*2*^	*R*^*2*^
298	0.0139	0.0743	0.8942	0.0377	0.0002	0.9998	0.0964	0.0303	0.9600
303	0.0148	0.1298	0.8348	0.0431	0.0010	0.9991	0.1165	0.0384	0.9562
308	0.0150	0.1448	0.8168	0.0441	0.0015	0.9986	0.1208	0.0386	0.9567
313	0.0157	0.2261	0.7485	0.0503	0.0024	0.9982	0.1455	0.0531	0.9510
318	0.0163	0.3075	0.7043	0.0557	0.0024	0.9985	0.1686	0.0718	0.9456
323	0.0190	1.2055	0.7312	0.0634	0.0046	0.9979	0.2039	0.0922	0.9458

**Figure 4:**
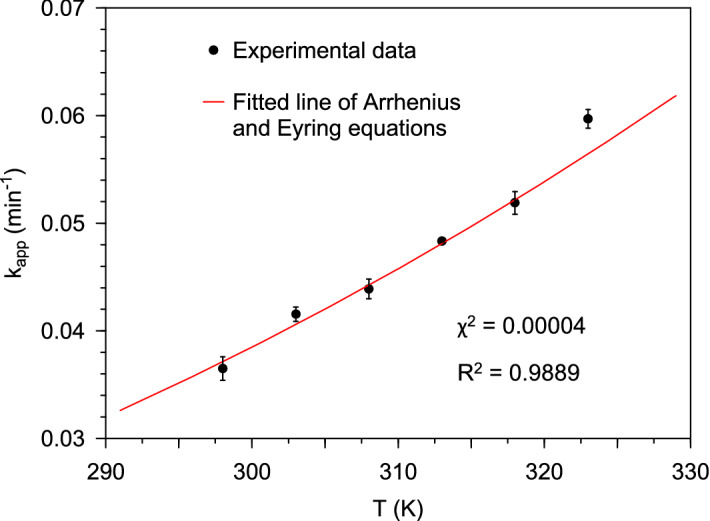
Effect of temperature if the apparent rate constant for Cr(VI) reduction by HCOOH over Pd@Polym-MNS.

Figure 4 shows the relationship between temperature and the apparent rate constant. An increase in temperature resulted in an increase in the value of the rate constant suggesting that the Cr(VI) conversion process was endothermic. Application of the exponential functions that represent the Arrhenius and Eyring equations to the thermodynamic data yielded good fit to the plot of the apparent rate constant versus temperature with a coefficient of determination (*R*^*2*^) value of 0.9889. From the parameters of the Arrhenius equation, the activation energy was found to be 13.4 kJ mol^−1^, which, suggested that the rate-controlling step of the overall Cr(VI) conversion process was physical in nature^6^. Therefore, the rate of mass transfer (diffusion), as opposed to the Cr(VI)-HCOOH redox reaction in the adsorbed phase, was the rate-controlling step.

Table 3 shows how the activation energy recorded in this work compared to that of other HCOOH-mediated Cr(VI) conversion processes on different Pd-loaded materials. There appeared to be a negative correlation between Pd loading and activation energy where the activation energy was lower for materials with higher Pd loading. Therefore, the low activation energy associated with the Pd@Polym-MNS was attributed to high Pd nanoparticle loading, which resulted in extensive surface coverage by the coated Pd. In turn, high Pd nanoparticle loading rendered the rate of the Cr(VI) conversion process so fast that the surface reaction step was not responsible for rate control.

**Table 3:** Activation energies of HCOOH-assisted Cr(VI) conversion on different Pd-loaded materials.

Material	Pd loading (wt %)	E_a_ (kJ mol^–1^)	References
Polyethersulfone bead-immobilized Pd nanoparticles	0.4	40.25	^17^
Viral-templated Pd nanocatalysts	-	27.3	^57^
Functionalized silica-supported Pd nanoparticles	1.4	25.9	^58^
Graphene oxide-supported Pd nanoparticles	1.2	24.5	^59^
*Macadamia* nutshell-supported Pd nanoparticles	13.25	13.4	This work

Application of the Eyring equation yielded the values of change in enthalpy, entropy, and Gibbs free energy of activation given in Table 4. The positive change in enthalpy of activation (*ΔH*^*‡*^) confirmed that the Cr(VI) conversion process was endothermic. The low value of *ΔH*^*‡*^ promoted the formation of the transition-state complex and was attributed to the catalytic action of the Pd coating of the Pd@Polym-MNS. The negative change in entropy of activation (*ΔS*^*‡*^) suggested that there was an improvement in the order of the reacting system, signifying the formation of a transition-state complex with fewer vibrational, rotational, and transitional states than the HCrO_4_^–^ and HCOOH species in the ground state^60^. In turn, this confirmed that the reaction that occurred on the surfaces of the Pd@Polym-MNS involved an associative mechanism in which the Cr(VI) and HCOOH species were adsorbed on the Pd@Polym-MNS surface^61,62^.

**Table 4:** Change in enthalpy, entropy, and Gibbs free energy of activation for HCOOH-mediated Cr(VI) conversion on Pd@Polym-MNS.

T (K)	*ΔG*^*‡*^ (kJ mol^–1^)	*ΔH*^*‡*^ (kJ mol^–1^)	*ΔS*^*‡*^ (J mol^–1^ K^–1^)
298	91.3	10.8	–270.0
303	92.6		
308	94.0		
313	95.3		
318	96.7		
323	98.0		

The positive values of the change in Gibbs free energy of activation (*ΔG*^*‡*^) demonstrated that the overall Cr(VI) conversion process was endergonic or non-spontaneous, which meant that the process lacked the natural tendency to occur without an external driving force in the form of an input of energy or another process coupled to it. The increase in *ΔG*^*‡*^ with increase in temperature shown in Table 4 suggested greater favorability of the Cr(VI) conversion process at lower temperature. Given that, as detailed in previous studies of the dehydrogenation of HCOOH on Pd-based catalysts^63,64^, the rate of hydrogen gas generation by adsorbed hydrogen atom recombination following the decarboxylation of HCOOH (Fig. 2) also increases with increase in temperature, favorability of the Cr(VI) conversion process at lower temperature could be attributed to diminution of the hydrogen recombination stage of HCOOH dehydrogenation. The practicability of the Pd@Polym-MNS facilitated Cr(VI) conversion was therefore attributed to the dehydrogenation of HCOOH, which provided the necessary driving force.

### Catalysis of porous basket reactor

#### Reactor efficacy, confirmation of Cr(III) species as the Cr(VI) conversion product, and Pd leaching study

**Figure 5:**
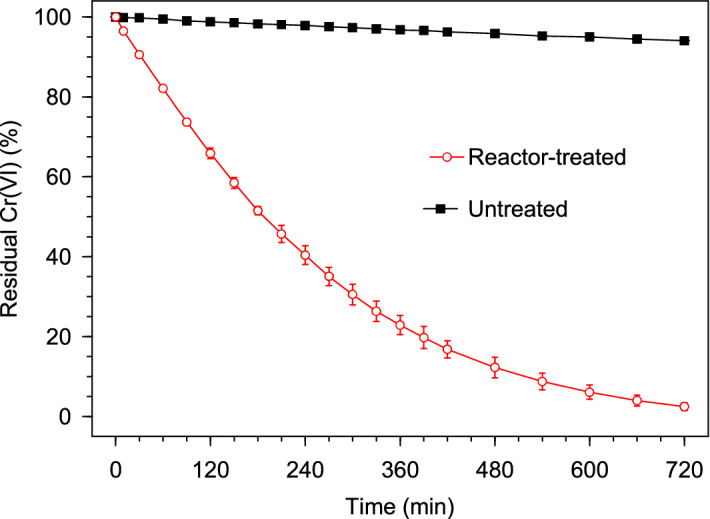
Evolution of the residual Cr(VI) in a mixed Cr(VI)-HCOOH solution treated with a Pd@Polym-MNS-packed reactor, and another that did not receive the treatment. Fixed experimental conditions: V = 5000 mL; Pd@Polym-MNS dose = 0.5 g L^–1^; T = 28 °C; [Cr(VI)]_0_ = 1.0 mM; [HCOOH]_0_ = 1000 mM.

The efficacy of the porous basket reactor mode was investigated by the treatment of a bulk (5000 mL) solution containing 1.0 mM Cr(VI) and 1000 mM HCOOH with a reactor packed with 2.5 g of Pd@Polym-MNS. As can be observed in Fig. 5, after 720 min (12 h) of submerging the reactor in the continuously stirred solution, the residual Cr(VI) had decreased to 2.5%, whereas for a similar mixed Cr(VI)-HCOOH solution that did not receive treatment with the reactor, the residual Cr(VI) only dropped to 94.1% emphasizing the need for the Pd@Polym-MNS to catalyze the Cr(VI) conversion process.

Overwhelming the reactor with large volume of Cr(VI) solution containing a relatively large initial Cr(VI) concentration enabled an output of Cr(III) ions sufficient for the generation of the color associated with the Cr(III) ions. Accordingly the occurrence of Cr(VI) reduction in the reactor-treated mixed with Cr(VI)-HCOOH solution was evidenced by a gradual change in the color of the solution from yellow to green and then blue. The final blue color was ascribed to the complex formed by the nascent Cr(III) ions and excess HCOOH, whereas the combination of the Cr(III)-HCOOH complex and unreacted Cr(VI) yielded the transient green color. To gain advantage from the extended period of exposure of the Pd@Polym-MNS to the acidic solution, an aliquot of the final solution was analyzed by ICP-OES whereupon no Pd was detected indicating that there was no leaching in the acidic conditions that prevailed during the Cr(VI) conversion process. The absence of Pd leaching in similar applications has previously been reported^17,55,65,66^.

#### Cr(VI) conversion performance

**Figure 6:**
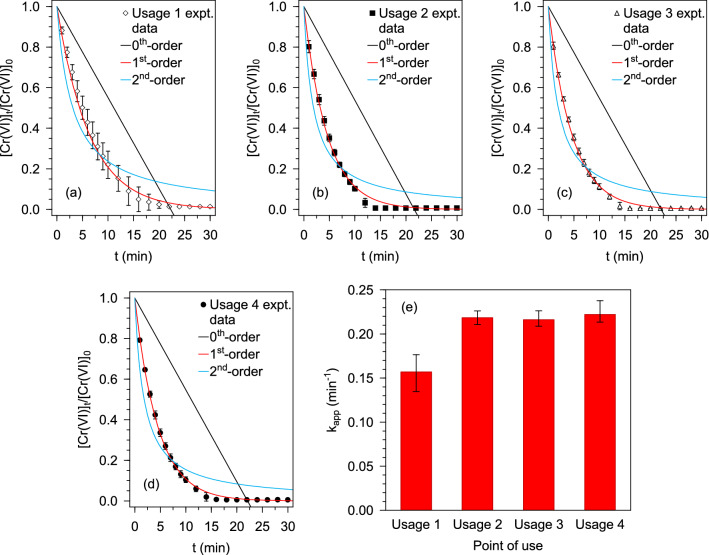
Kinetic plots of remaining fraction of Cr(VI) versus reaction time for Cr(VI) reduction by HCOOH over Pd@Polym-MNS at different points of consedutive reactor use: **(a)** Usage1, **(b)** Usage 2, **(c)** Usage 3, **(d)** Usage 4. **(d)** Comparison of the corresponding pparent rate constants. Fixed experimental conditions: V = 500 mL; Pd@Polym-MNS dose = 5.0 g L^–1^; T = 60 °C; [Cr(VI)]_0_ = 2.0 mM; [HCOOH]_0_ = 2000 mM.

In order to evaluate the performance of the Pd@Polym-MNS loaded reactor with respect to the reduction of Cr(VI) using formic acid, and to facilitate comparison with previously reported Pd-based catalyst, a reactor specimen containing 2.5 g of Pd@Polym-MNS was used to consecutively treat four 500 mL aliquots of mixed Cr(VI)-HCOOH solutions initially containing 2.0 mM Cr(VI) and 2000 mM HCOOH. Between usages, the basket was stirred in air for 30 min. The obtained kinetic plots of *[Cr(VI)]*_*t*_/*[Cr(VI)]*_*0*_ versus *t*, which illustrate that pseudo-second-order kinetic model provided best fit to the experimental data in each instance, are presented in Fig. 6a–d (*R*^*2*^ = 0.9894, 0.9949, 0.9966, 0.9982 for the first, second, third and fourth usage, respectively). The apparent rate constant as per the pseudo-second-order kinetic model was plotted against the instance of use as shown in Fig. 6e, which depicts an increase in the apparent rate constant from 0.157 min^–1^ upon the first usage to 0.218 min^–1^ found for the second usage. Since the redox reaction between the Cr(VI) and HCOOH occurred in the adsorbed phase on the surfaces of the Pd@Polym-MNS, the rate reaction was dependent on the quantities of adsorbed Cr(VI) species and HCOOH. The first usage involved the establishment of the state of adsorption equilibrium, which then presented the highest concentration of adsorbed Cr(VI) ions. Therefore, the lower pre-equilibrium concentration of adsorbed Cr(VI) yielded lower rates of Cr(VI) conversion on the Pd@Polym-MNS surface.

Moreover, the highest degree of effervescence, which was ascribed to the release of hydrogen gas (H_2_) in addition to CO_2_, was witnessed during the first usage. We, therefore, posited that in the initial stages of the first usage, the lighter, hence more mobile, HCOOH molecules with a formula mass of 46.03 amu were more rapidly adsorbed onto the Pd@Polym-MNS surface in comparison to the HCrO_4_^–^ and Cr_2_O_7_^2–^ ions with formula masses of 117.00 amu and 215.99 amu, respectively. Consequently, the accumulation of adsorbed hydrogen atoms from the decarboxylation of HCOOH in the presence of a relatively smaller quantity of adsorbed Cr(VI) ions to participate in redox reactions promoted the formation and desorption of H_2_ thereby increasing the extent of effervescence in the first usage.

The evened out values of the apparent rate constant values recorded as 0.218 min^–1^, 0.216 min^–1^ and 0.222 min^–1^ for the second, third and fourth usages, respectively, indicated that at the end of the first usage the quantity of adsorbed Cr(VI) had increased to the equilibrium level that resulted in a constant rate of the redox reaction with adsorbed hydrogen atoms. The participation of adsorbed hydrogen atoms in the reduction of adsorbed Cr(VI), as opposed to combination thereby forming H_2_, was substantiated by the observation of lower extents of effervescence. Furthermore, the plateauing of the apparent rate constant values indicated the stability, hence reusability, of the Pd@Polym-MNS.

#### Effect of interfering anions

**Figure 7:**
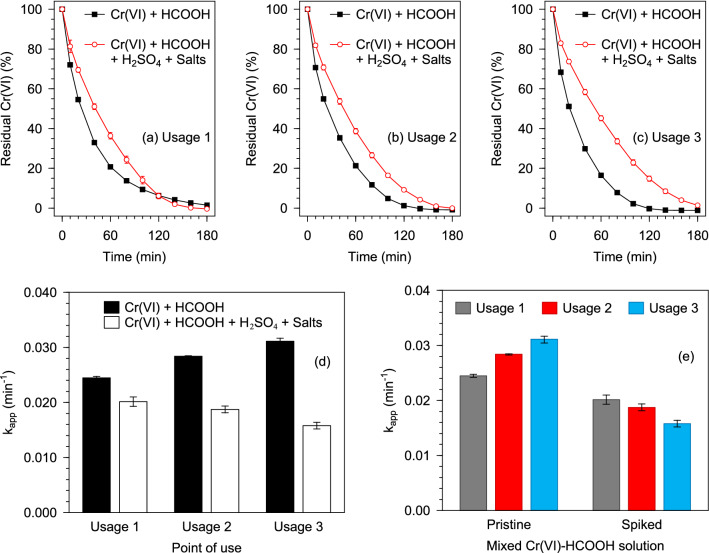
Evolution of the residual Cr(VI) in pristine and spiked mixed Cr(VI)-HCOOH solution treated with a Pd@Polym-MNS-packed reactor at different points of consedutive reactor use: **(a)** Usage1, **(b)** Usage 2, **(c)** Usage 3. **(d,e)** Comparison of the corresponding apparent rate constants. Fixed experimental conditions: V = 500 mL; Pd@Polym-MNS dose = 2.0 g L^–1^; T = 50 °C; [Cr(VI)]_0_ = 1.0 mM; [HCOOH]_0_ = 200 mM; [SO_4_^2–^]_0_ = 1000 mM; [Cl^–^]_0_ = 200 mM; [NO_3_^–^]_0_ = 300 mM.

To assess the influence of anionic interference on the activity of the Pd@Polym-MNS, two identical reactors were used to consecutively treat three mixed Cr(VI)-HCOOH solutions. The first reactor was used on pristine solutions with no spike reagents whereas the second was applied on solutions spiked with H_2_SO_4_ (1000 mM), NaCl (200 mM), and NaNO_3_ (300 mM) in an attempt to simulate the conditions of electroplating wastewater^67^. After each treatment, the reactor was flushed under running tap water and then soaked in 250 mL of fresh tap water for 30 min before the commencement of the next usage. The catalytic activity of the Pd@Polym-MNS in each matching usage was compared by plotting the remaining fraction of Cr(VI) in the solution as presented in Fig. 7a–c. Irrespective of the composition of the mixed Cr(VI)-HCOOH solution, less than 2% of the initial Cr(VI) remained in the solution after 180 min of contact with the Pd@Polym-MNS in all three instances of usage. However, for the first two-thirds of the first usage (up to 120 min) as well as the entirety of the second and third usages, the residual Cr(VI) proportion at a particular time was higher in the spiked solutions than in the pristine solutions, which is reflected by the comparison of the corresponding apparent rate constant values derived from fitting the pseudo-second-order model equation (Fig. 7d). In addition to the adsorption of Cr(VI) ions onto the Pd@Polym-MNS surface, there was competitive uptake of the co-existing SO_4_^2–^, Cl^–^ and NO_3_^–^ ions from the spiked mixed Cr(VI)-HCOOH solutions. Consequently, the quantity of Cr(VI) ions adsorbed from the spiked solutions was lower than that from the pristine solutions hence the lower rated rates of Cr(VI) conversion signified by the lower apparent rate constants.

Since the spike reagents were present in large concentrations, the Pd@Polym-MNS surface became extensively covered by SO_4_^2–^, Cl^–^ and NO_3_^–^ ions during the first usage. Therefore, the majority of the adsorption sites that were available for the uptake of Cr(VI) ions in the subsequent usages were those from which nascent Cr(III) ions were desorbed. However, in the prevailing conditions of high SO_4_^2–^, Cl^–^ and NO_3_^–^ ion concentration, the greater part of the vacated adsorption sites proceeded to be occupied by the co-existing ions. Ultimately, the quantity of adsorbed Cr(VI) ions continuously decreased with consecutive use of the Pd@Polym-MNS as indicated by the continuous decrease in the rate constant for Cr(VI) conversion in the spiked solutions as depicted by Fig. 7e. Treatment of the pristine solutions yielded a successive increase in the value of the rate constant indicating successive increases in the rate of Cr(VI) conversion thereby suggesting that succeeding usages involved larger quantities of adsorbed Cr(VI) ions. Successive increase in the quantity of adsorbed Cr(VI) ions pointed to incomplete coverage of the Pd@Polym-MNS surface by the Cr(VI) ions. Therefore, additional uptake of Cr(VI) ions on vacant sites on the Pd@Polym-MNS surface upon the introduction of fresh mixed Cr(VI)-HCOOH solution in the next usage resulted in an increase in the quantity of adsorbed Cr(VI) ions available for redox reaction with active hydrogen atoms adsorbed on the Pd surface, which, in turn, increased the rate of Cr(VI) conversion.

#### Response surface methodological modeling of reactor performance

The results obtained from the central composite design-based experiments are summarized in Table 5 wherein the responses of the center point experiment (run numbers 25–30) exhibited a coefficient of variation of 4.9%, which indicated a desirable level of reproducibility and accuracy of the experiments. The model equation relating the apparent rate constant (*k*_*app*_) to the initial Cr(VI) concentration (*[Cr(VI)]*_*0*_), initial HCOOH concentration (*[HCOOH]*_*0*_), Pd@Polym-MNS dose (*Dose*), and temperature (*T*) was found to be as follows:

20 $$k_{app} = {-} \, 0.00{61536 } + \, 0.00{59316}\left[ {Cr\left( {VI} \right)} \right]_{0} + \, 0.0000{46}0\left[ {HCOOH} \right]_{0} {-} \, 0.00{218}0{1}Dose{-} \, 0.000{14}0{9}T{-} \, 0.0000{192}\left[ {Cr\left( {VI} \right)} \right]_{0} \left[ {HCOOH} \right]_{0} {-} \, 0.000{625}0\left[ {Cr\left( {VI} \right)} \right]_{0} Dose{-} \, 0.0000{168}\left[ {Cr\left( {VI} \right)} \right]_{0} T + \, 0.0000{159}\left[ {HCOOH} \right]_{0} Dose + \, 0.000000{6}[HCOOH]_{0} T + \, 0.000{24}0{1}DoseT {-} \, 0.000{8}0{71}\left[ {Cr\left( {VI} \right)} \right]_{0}^{{2}} {-} \, 0.000000{1}\left[ {HCOOH} \right]_{0}^{2} {-} \, 0.0000{619}Dose^{{2}} + \, 0.000000{3}T^{2}$$

**Table 5:** Central composite design matrix and experimental results for HCOOH-mediated Cr(VI) conversion on Pd@Polym-MNS.

Run no	[Cr(VI)]_0_ (mM)	[HCOOH]_0_ (mM)	Dose (g L^–1^)	T (°C)	Response, k (min^–1^)	
					Observed	Predicted
1	1.5	100	1	25	0.00459	0.00379
2	1.5	100	1	45	0.00794	0.00688
3	1.5	100	3	25	0.01202	0.01224
4	1.5	100	3	45	0.02325	0.02494
5	1.5	300	1	25	0.00577	0.00680
6	1.5	300	1	45	0.01149	0.01235
7	1.5	300	3	25	0.02140	0.02163
8	1.5	300	3	45	0.03708	0.03679
9	2.5	100	1	25	0.00236	0.00353
10	2.5	100	1	45	0.00689	0.00628
11	2.5	100	3	25	0.01197	0.01074
12	2.5	100	3	45	0.02325	0.02310
13	2.5	300	1	25	0.00478	0.00271
14	2.5	300	1	45	0.00727	0.00793
15	2.5	300	3	25	0.01435	0.01628
16	2.5	300	3	45	0.03068	0.03111
17	0.5	200	2	35	0.01827	0.01790
18	3	200	2	35	0.01116	0.01149
19	2	50	2	35	0.00796	0.00925
20	2	500	2	35	0.01655	0.01601
21	2	200	0.5	35	0.00195	0.00326
22	2	200	5	35	0.03899	0.03844
23	2	200	2	15	0.00640	0.00642
24	2	200	2	55	0.02485	0.02433
25	2	200	2	35	0.01574	0.01527
26	2	200	2	35	0.01592	0.01527
27	2	200	2	35	0.01688	0.01527
28	2	200	2	35	0.01598	0.01527
29	2	200	2	35	0.01559	0.01527
30	2	200	2	35	0.01445	0.01527

**Figure 8:**
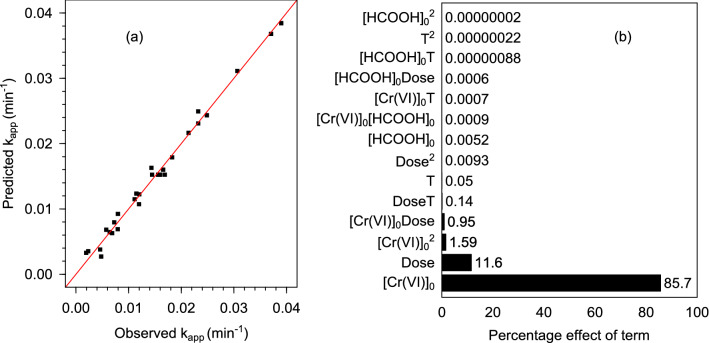
**(a)** Parity plot of observed versus predicted apparent rate constant values, and **(b)** Pareto plot of percentage effects for the developed response surface methodology-based model for Cr(VI) reduction by HCOOH in a Pd@Polym-MNS packed in a porous basket reactor.

The agreement of the observed apparent rate constant values with those predicted by Eq. (20) was exhibited by the distribution of the data about the x = y line as shown in Fig. 8a. Using the coefficients of the model equation, the percentage effect of each term on the value of the predicted rate constant, *P*_*i*_ (%), was calculated using Eq. (21) expressed as:

21 $${\text{P}}_{\text{i}}\, = \,\frac{{\text{b}}_{\text{i}}^{2}}{\sum {\text{b}}_{\text{i}}^{2}}\,{ \times \, 100\%}$$

where *b*_*i*_ (dimensionless) coefficient of term *i*^68^. As shown in Fig. 8b the initial Cr(VI) concentration and Pd@Polym-MNS, accounting for 85.7% and 11.6%, respectively, produced the greatest effect on the rate of Cr(VI) conversion, demonstrating that the reaction heavily depended on the adsorption of the Cr(VI) ions on the Pd@Polym-MNS.

Analysis of variance (ANOVA), based on testing the null hypothesis that the developed regression model fitted the data well against the alternative hypothesis that the model did not provide a good fit to the experimental data, was conducted (Table 6). The *F*-value of the regression procedure, which was found to be 98.95, was higher than the critical F-value at a significance level of 0.05 (2.424). Therefore, statistical significance, corresponding to the lack of support for the null hypothesis, was reached. The coefficient of determination (*R*^*2*^) was 0.9893 indicating a good correlation between observed and model-predicted values of the first-order rate constant as shown in Fig. 8a. Accordingly, the model was deemed sufficient for the generation of response surface plots and subsequent analysis of the combined influence of the input variables.

**Table 6:** Analysis of variance for the regression-modeled rate constant for HCOOH-mediated Cr(VI) conversion on Pd@Polym-MNS.

Variance source	Sum of squares	Degree of freedom	Mean square	F-value	F_crit_
Regression	0.002547	14	0.0001819	98.95	2.424
Residues	0.000028	15	0.0000018		
Total	0.002575	29			

**Figure 9:**
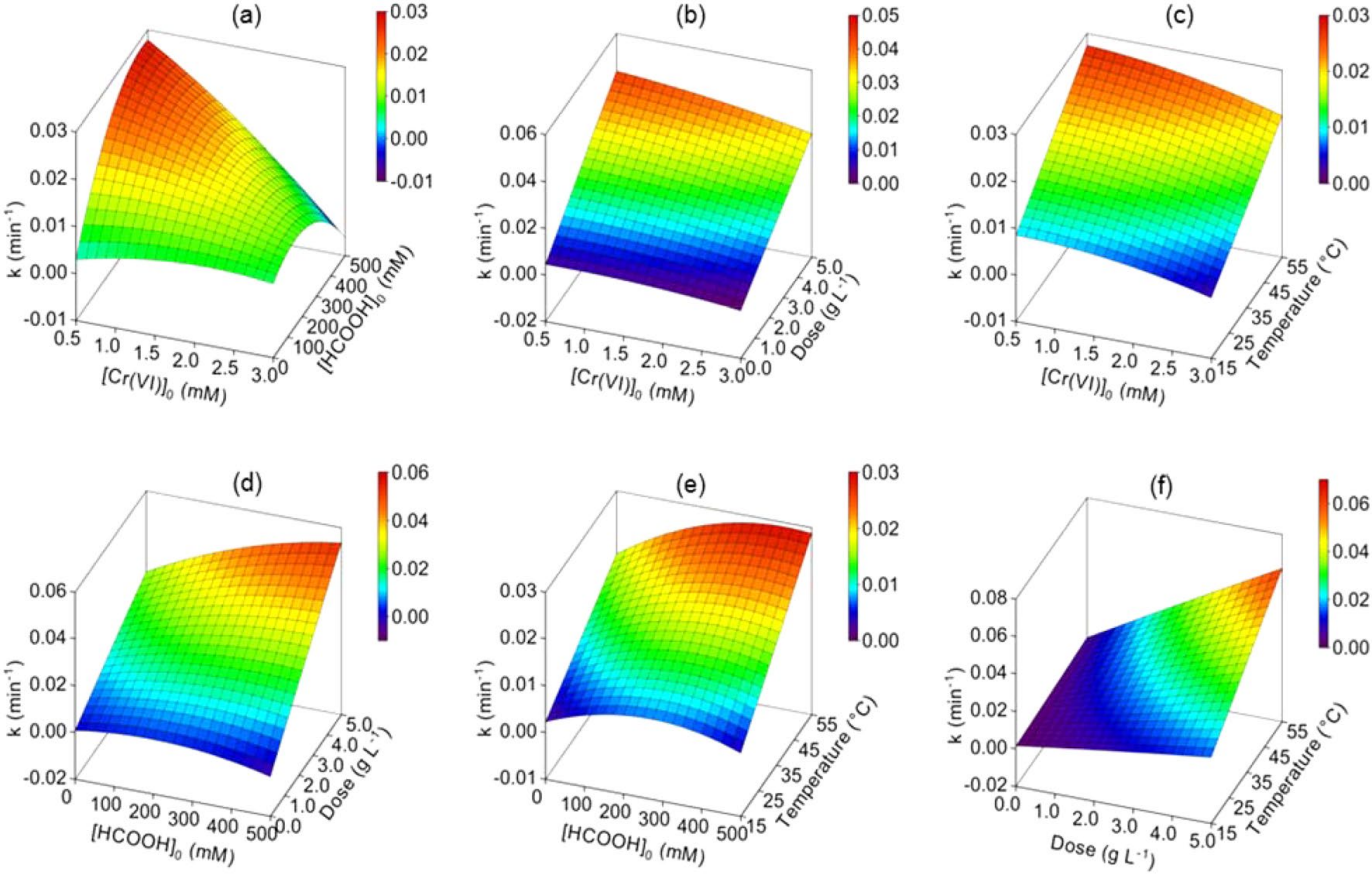
Response surface plots showing the combined effects of **(a)** initial Cr(VI) concentration and initial HCOOH concentration, **(b)** initial Cr(VI) concentration and Pd@Polym-MNS dose, **(c)** initial Cr(VI) concentration and temperature, **(d)** initial HCOOH concentration and Pd@Polym-MNS dose, **(e)** initial HCOOH concentration and temperature, and **(f)** Pd@Polym-MNS dose and temperature on Cr(VI) reduction by HCOOH over Pd@Polym-MNS packed in a porous basket reactor.

The response surface plots showing the influences of different combinations of the input variables on the rate constant for Cr(VI) conversion on the Pd@Polym-MNS are presented in Fig. 9. Varying the Cr(VI) concentration at low HCOOH concentration had little impact on the rate constant (Fig. 9a). However, at high HCOOH concentration, the rate constant decreased with increasing Cr(VI) concentration. At any given Cr(VI) concentration, the value of the rate constant increased with increasing HCOOH concentration. The greatest impact of increasing the HCOOH concentration was at low Cr(VI) concentration where the value of the rate constant increased more rapidly. Therefore, the combined effect of the initial concentrations of Cr(VI) and HCOOH had the greatest impact at low Cr(VI) concentration and high HCOOH concentration.

While as shown by Fig. 9b,c, varying the initial Cr(VI) concentration at any Pd@Polym-MNS dose or temperature had no impact on the rate constant, neither the increase in Pd@Polym-MNS dose nor temperature at any Cr(VI) concentration yielded higher values of the rate constant. Therefore, neither the combination of initial Cr(VI) concentration and Pd@Polym-MNS dose nor initial Cr(VI) concentration and the temperature had a mutual influence on the rate of Cr(VI) conversion. According to Fig. 9d, varying the initial HCOOH concentration at low Pd@Polym-MNS dose had little effect on the value of the rate constant. Conversely, at high Pd@Polym-MNS dose, increasing the initial HCOOH concentration resulted in an increase in the rate constant. Although increasing the Pd@Polym-MNS dose at any initial HCOOH concentration resulted in the higher rate constant values, the increase at lower HCOOH concentration was less rapid than at higher HCOOH concentration. Therefore, the initial HCOOH concentration and Pd@Polym-MNS dose had a combined effect on the Cr(VI) conversion rate whereupon high values of both input variables resulted in amplified values of the rate constant.

Although increasing the temperature at any HCOOH concentration yielded an increase in the value of the rate constant, changing the initial HCOOH concentration at a given temperature had a minor impact on the rate constant (Fig. 9e). Therefore, the combination of initial HCOOH concentration and the temperature had little influence on the rate of Cr(VI) conversion. Though, as per Fig. 9f, increasing the Pd@Polym-MNS dose at a low temperature slightly increased the value of the rate constant, a similar increase in the Pd@Polym-MNS dose at higher temperature resulted in a greater increase in the rate constant value. Likewise, the increase in the value of the rate constant with increasing temperature was more intense at higher Pd@Polym-MNS dose indicating the existence of a mutual influence of these input variables.

### Comparison of reactor performance with that of suspended Pd@Polym-MNS and other catalysts

**Figure 10:**
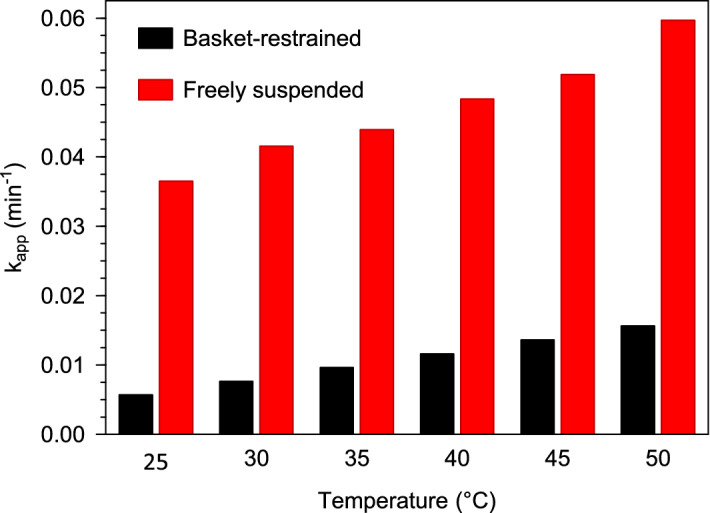
Comparison of apparent rate constant values obtained at different temperatures for Cr(VI) reduction by HCOOH over freely suspended Pd@Polym-MNS to predicted values for reduction in a Pd@Polym-MNS packed in a porous basket reactor.

Owing to the adverse effects of aggregation, which results in less effective exposure of the Pd@Polym-MNS packed in the basket reactor to the mixed Cr(VI)-HCOOH solution, it was anticipated that the performance of the basket-restrained Pd@Polym-MNS would be lesser than that of the freely suspended Pd@Polym-MNS. In order to facilitate comparison of the performance of the Pd@Polym-MNS in the freely suspended and basket-restrained modes, Eq. (20), the equation of the model as derived by response surface methodology, was used for the prediction of the apparent rate constant values that the basket reactor would yield under the conditions of the thermodynamic parameter determination experiments in which freely suspended Pd@Polym-MNS was used. Figure 10 compares the experimentally determined apparent rate constant values to the predictions of the RSM-derived model at the different incubation temperatures used upon the thermodynamic parameter determination experiments.

At each of the five temperatures, there was an evident discrepancy between the two apparent rate constants. The attenuated apparent rate constant values predicted for the basket-restrained Pd@Polym-MNS were 73.8% to 84.4% lower than the values recorded when the freely suspended Pd@Polym-MNS was used. However, it is noteworthy that, as shown in Table 7, the highest apparent rate constant value recorded as 0.222 min^–1^ for the basket-restrained Pd@Polym-MNS was higher than previously reported values noted for Cr(VI) reduction processes catalyzed by a few Pd-based materials including polyethersulfone bead-immobilized Pd nanoparticles and Pd nanocrystals supported on reduced graphene oxide. The majority of examples listed in Table 7 illustrated comparable performance to that of the Pd@Polym-MNS.

**Table 7:** Rate constant values of HCOOH-assisted Cr(VI) conversion on different Pd-loaded materials.

Catalyst	k_app_ (min^–1^)	Reference
Polyethersulfone bead-immobilized Pd nanoparticles	0.167	^17^
PdCu nanoboxes	0.274	^18^
Amino-functionalized Pd nanowires	0.282	^69^
Pd nanocrystals supported on reduced graphene oxide	0.0785	^70^
Polyurea microsphere immobilized Pd	0.123	^71^
Pd nanoparticles immobilized on procyanidin-grafted eggshell membrane	0.133	^72^
Magnetic mesoporous carbon doped with Pd nanoparticles	0.267	^73^
Pd decorated carbon nanotubes	0.310	^74^
Fe_3_O_4_/Pd nanoparticles encapsulated in nitrogen-doped carbon	0.300	^75^
*Macadamia* nutshell-supported Pd nanoparticles (Pd@Polym-MNS)	0.222	This work

## Conclusions

Zerovalent palladium impregnated in polymer-grafted *Macadamia* nutshell biomass packed in a porous basket reactor to enhance the ease of recovery for recurrent use provides a reactor that adsorbs aqueous Cr(VI) species and formic acid molecules. Thereafter, the formic acid adsorbed on the palladium decomposes and generates reactive hydrogen species that undergo a redox reaction with the adsorbed Cr(VI) species resulting in the conversion of Cr(VI) to Cr(III). The naturally acidic conditions that prevail in the presence of the formic acid ensure that a large proportion of the nascent Cr(III) ions is desorbed from the catalyst surface thereby preventing catalyst deactivation. According to weighted Pareto analysis of the coefficients of a fitted response surface methodological model that relates the apparent rate constant of the Cr(VI) conversion process to initial Cr(VI) concentration, initial formic acid concentration, catalyst dosage and solution temperature, which have the highest impact on the performance of the porous basket reactor, initial Cr(VI) concentration and catalyst dosage account for the largest proportion of the observed changes in the value of the apparent rate constant. The results of this study demonstrate that simple reactors with competitive catalytic activity can be constructed using wire mesh that not only allows easy separation of the catalyst from the treated solution but also helps prevent damage of the catalyst particles by collisions amongst themselves, and with stirrer blades and container walls. The study sets the pace for the development of similarly built reactors with modifications that minimize catalyst aggregation and enhance the exposure of the restrained catalyst to the solution requiring treatment.
